# Post-treatment with PT302, a long-acting Exendin-4 sustained release formulation, reduces dopaminergic neurodegeneration in a 6-Hydroxydopamine rat model of Parkinson’s disease

**DOI:** 10.1038/s41598-018-28449-z

**Published:** 2018-07-16

**Authors:** Shuchun Chen, Seong-Jin Yu, Yazhou Li, Daniela Lecca, Elliot Glotfelty, Hee Kyung Kim, Ho-Il Choi, Barry J. Hoffer, Nigel H. Greig, Dong Seok Kim, Yun Wang

**Affiliations:** 1 Center for Neuropsychiatric Research, National Health Research Institutes, Zhunan, Taiwan; 2 Graduate Institute of Applied Science and Engineering, Fu-Jen Catholic University, New Taipei, Taiwan; 3 Drug Design and Development Section, Translational Gerontology Branch, Intramural Research Program, National Institute on Aging, National Institutes of Health, Baltimore, MD USA; 4 Peptron Inc., Yuseong-gu, Daejeon, Republic of Korea; 5 Department of Neurosurgery, Case Western Reserve University School of Medicine, Cleveland, OH USA

## Abstract

We previously demonstrated that pretreatment with Exendin-4, a glucagon-like peptide-1 (GLP-1) receptor agonist, reduces 1-methyl-4-phenyl-1,2,3,6-tetrahydropyridine (MPTP) –mediated dopaminergic neurodegeneration. The use of GLP-1 or Exendin-4 for Parkinson’s disease (PD) patients is limited by their short half-lives. The purpose of this study was to evaluate a new extended release Exendin-4 formulation, PT302, in a rat model of PD. Subcutaneous administration of PT302 resulted in sustained elevations of Exendin-4 in plasma for >20 days in adult rats. To define an efficacious dose within this range, rats were administered PT302 once every 2 weeks either before or following the unilaterally 6-hydroxydopamine lesioning. Pre- and post-treatment with PT302 significantly reduced methamphetamine–induced rotation after lesioning. For animals given PT302 post lesion, blood and brain samples were collected on day 47 for measurements of plasma Exendin-4 levels and brain tyrosine hydroxylase immunoreactivity (TH-IR). PT302 significantly increased TH-IR in the lesioned substantia nigra and striatum. There was a significant correlation between plasma Exendin-4 levels and TH-IR in the substantia nigra and striatum on the lesioned side. Our data suggest that post-treatment with PT302 provides long-lasting Exendin-4 release and reduces neurodegeneration of nigrostriatal dopaminergic neurons in a 6-hydroxydopamine rat model of PD at a clinically relevant dose.

## Introduction

Parkinson’s disease (PD) is the second most common neurodegenerative disorder after Alzheimer’s disease, and afflicts approximately 1% of the population worldwide over the age of 60 years^1^. The number of PD patients is expected to more than double by 2040^2,3^, and, although there are many symptomatic treatments for PD, none appear to have a significant effect on disease progression. The primary mechanism underpinning most approved PD drugs is to augment the dopaminergic transmission. Levodopa is the most commonly prescribed drug for PD, but its use and that of other dopaminergic agonists are limited by side effects and complications^4^. Thus, there is a need to develop non-dopaminergic pharmacological treatments for PD and, in particular, neuroprotective or disease modifying therapies that can slow or halt disease progression.

Metabolic syndrome is increasingly recognized as a key factor in PD^5–7^, together with growing evidence that impaired insulin signaling plays a role in PD pathogenesis^8–10^. Supporting this, numerous, albeit not all, epidemiological studies suggest that diabetes is associated with increased risk for PD^11–13^. Furthermore, dysfunctional neuronal insulin signaling has been demonstrated in toxin-induced as well as high fat diet-induced animal models of PD and appears to exacerbate PD-associated impairments^14–16^. There is also evidence of dysregulated neuronal insulin signaling in human PD and PD dementia^8^. Given that efficient insulin signaling is critical for neuronal survival, the loss of this important pathway may thereby result in neurodegeneration. Neuropathological studies of PD patients indicate a dense localization of insulin receptors in dopaminergic neurons within the substantia nigra pars compacta^17^, and their decline, as evaluated by messenger RNA or immunoreactivity (IR), is associated with a loss of tyrosine hydroxylase (TH) messenger RNA, the rate-limiting enzyme involved in dopamine synthesis^18^. In parallel with this is evidence from animal models, in which the development of insulin resistance coincides with a decline in surface dopamine transporter levels in striatum^19^, reduced insulin-evoked striatal dopamine release^20^ and a decreased dopamine turnover^15,20,21^. Overcoming aberrant insulin signaling can potentially be clinically achieved, and hence could represent a new treatment approach for neurodegenerative disorders.

Incretins, such as glucagon-like peptide 1 (GLP-1) and glucose-dependent insulinotropic polypeptide (GIP), are gastrointestinal peptides that, following their release from the L and K cells of the gastrointestinal tract in response to food ingestion, regulate pancreatic β-cell insulin release. Their receptors, GLP-1R and GIP-R, respectively, are expressed within the brain, in addition to pancreatic β-cells^22–24^. The presence of the GLP-1R, in particular, has been demonstrated in the midbrain and striatum^25^. The activation of GLP-1R on neurons induces potent neurotrophic and neuroprotective actions in cellular and animal models of neural injury and neurodegeneration^26^, including models of PD^27,28^.

The use of GLP-1 and GIP as potential treatments for diabetes mellitus type 2 (T2DM) or PD is greatly limited by their short half-lives, of less than 5 min^29^. However, long-acting analogues that include Exendin-4 (Exenatide), a GLP-1R agonist originally isolated from the saliva of the lizard Heloderma suspectum and now synthesized, are approved for the treatment of T2DM^30,31^ and have shown promising actions in PD animal models^26^ and clinical trials of PD patients^32,33^. We previously demonstrated that pretreatment with Exendin-4 protected primary ventromesenchephalic neurons from 6-hydroxydopamine (6-OHDA) lesioning. Exendin-4 also mitigated the loss of dopaminergic neurons, preserved dopamine levels in the mouse substantia nigra, and improved behavioral function of mice receiving 1-methyl-4-phenyl-1,2,3,6-tetrahydropyridine (MPTP)^26^. Recent reports have indicated that PD patients taking Exendin-4 for one year had better motor skills than those on placebo^34,35^. However, following subcutaneous administration, Exendin-4 has an elimination half-life of 2.4 h and thus requires administration twice daily^31^. Such dosing is not optimal for clinical utilization involving patient populations with either movement disorders or cognitive deficits.

To maintain therapeutic levels in plasma, Exendin-4 has been formulated for release from biodegradable microspheres by using the polymer, poly-(D,L-lactide-co-glycolide) (PLGA), to provide once weekly subcutaneous dosing (*Bydureon*)^31^. The recent development of PT302 likewise employs PLGA, but the development of a proprietary ultrasonic spray drying process together, provides a formulation that can be injected through a smaller needle to minimize injection pain, suppresses the initial release burst of Exendin-4 and yielded a once every two-week (biweekly) medication in man^36^. In this study, we examined the protective effect of this Exendin-4 formulation, termed PT302, in a 6-OHDA rat model of PD. A time- and dose-dependent pharmacokinetic evaluation of PT302 was undertaken to provide sustained plasma Exendin-4 levels of relevance for human use. Thereafter, an efficacy dose-finding study was performed in adult rats challenged with a unilateral 6-OHDA lesion in the left medial forebrain bundle by first evaluating PT302 as pretreatment to delineate a dose of PT302 and second for subsequent post-treatment after unilateral 6-OHDA lesioning.

We now report this post-treatment paradigm with PT302 significantly reduced methamphetamine (meth)-induced rotational behavior, increased the survival of dopamine neurons in the lesioned substantia nigra, and preserved TH-IR in the lesioned striatum. Our data hence indicates that PT302 has a neuroprotective effect for nigrostriatal dopaminergic neurons in a 6-OHDA rat model of PD, and provides an efficient method to support sustained plasma levels of Exendin-4 through biweekly, or less frequent, subcutaneous administration for future human studies.

## Results

### Plasma Exendin-4 levels are time-dependently maintained by PT302 sustained release administration

**Figure 1:**
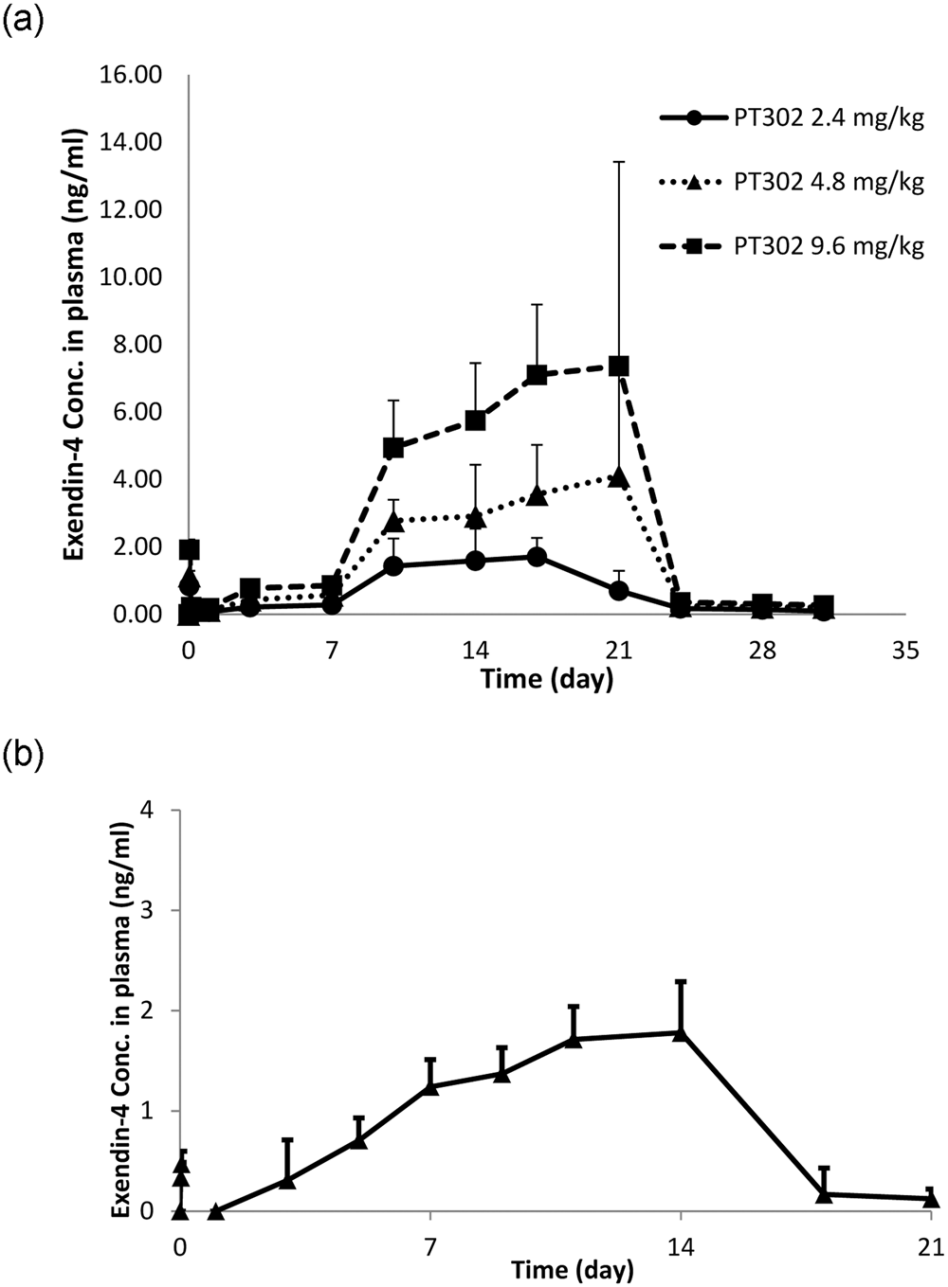
A single PT302 s.c. administration provides sustained levels of Exendin-4 in plasma, whose Cmax and AUC is increased linearly in a dose-dependent manner. PT302, a sustained release formulation of Exendin-4, contains a mixture of polymers (98%) and Exendin-4 (2%). Time-dependent plasma levels of Exendin-4 were quantified by ELISA after (**a**) injection of three doses of s.c. PT302 (equivalent to 2.4, 4.8 and 9.6 mg/kg Exendin-4) into separate cohorts of 9 week old male Sprague-Dawley rats (n = 6 per group, blood sampled at 0, 0.5, and 1 hr after injection, as well as on days 1, 3, 5, 7, 9, 11, 14, 18, 21, and 26 post-injection). (**b**) Based on these pharmacokinetics studies, a PT302 dose equivalent to 2.0 mg/kg Exendin-4 was then evaluated in a separate series of alike rodents.

Illustrated in Fig. 1 are the time-dependence curves of Exendin-4 released into plasma following a single subcutaneous administration of PT302 at increasing doses (2.4 mg/kg, 4.8 mg/kg, and 9.6 mg/kg), with samples collected at 0 to 1 hr and up to 31 days post-injection. Following an initial regulated release of Exendin-4, plasma peptide concentration time-dependently increased and reached steady-state levels from approximately 10 days onward. Exendin-4 achieved a maximal release concentration (Cmax) of 2.23 ng/ml at 14.8 days (Tmax) providing a time-dependent concentration (area under the curve, AUC) of 21.13 ng.d/ml for the 2.4 mg/kg dose. A Cmax of 5.21 ng/ml, with a Tmax of 16.17 days, and AUC of 49.46 ng.d/ml was achieved for the 4.8 mg/kg dose; and a Cmax of 9.42 ng/ml, with a Tmax of 17.17 days, and AUC of 87.14 ng.d/ml was obtained for the 9.6 mg/kg dose. This data indicates that elevations in PT302 dose were accompanied by a linear increase in Cmax and AUC of Exendin-4 in plasma, with the maintenance of Tmax. Notable, the initial regulated release of Exendin-4 reached a concentration in plasma lower than the final Cmax.

On the basis of the above dose- and time-dependent evaluation, a PT302 dose of 2.0 mg/kg was selected for further studies. Its administration as a single subcutaneous injection (s.c.) to rats similarly provided sustained levels of Exendin-4 in plasma (Fig. 1b) with a Cmax of 1.85 ng/ml, a Tmax of 12.5 days, and an AUC of 18.55 ng.d/ml.

### Pre-treatment with PT302 reduces meth-mediated rotation in the 6-OHDA rat model of PD

**Figure 2:**
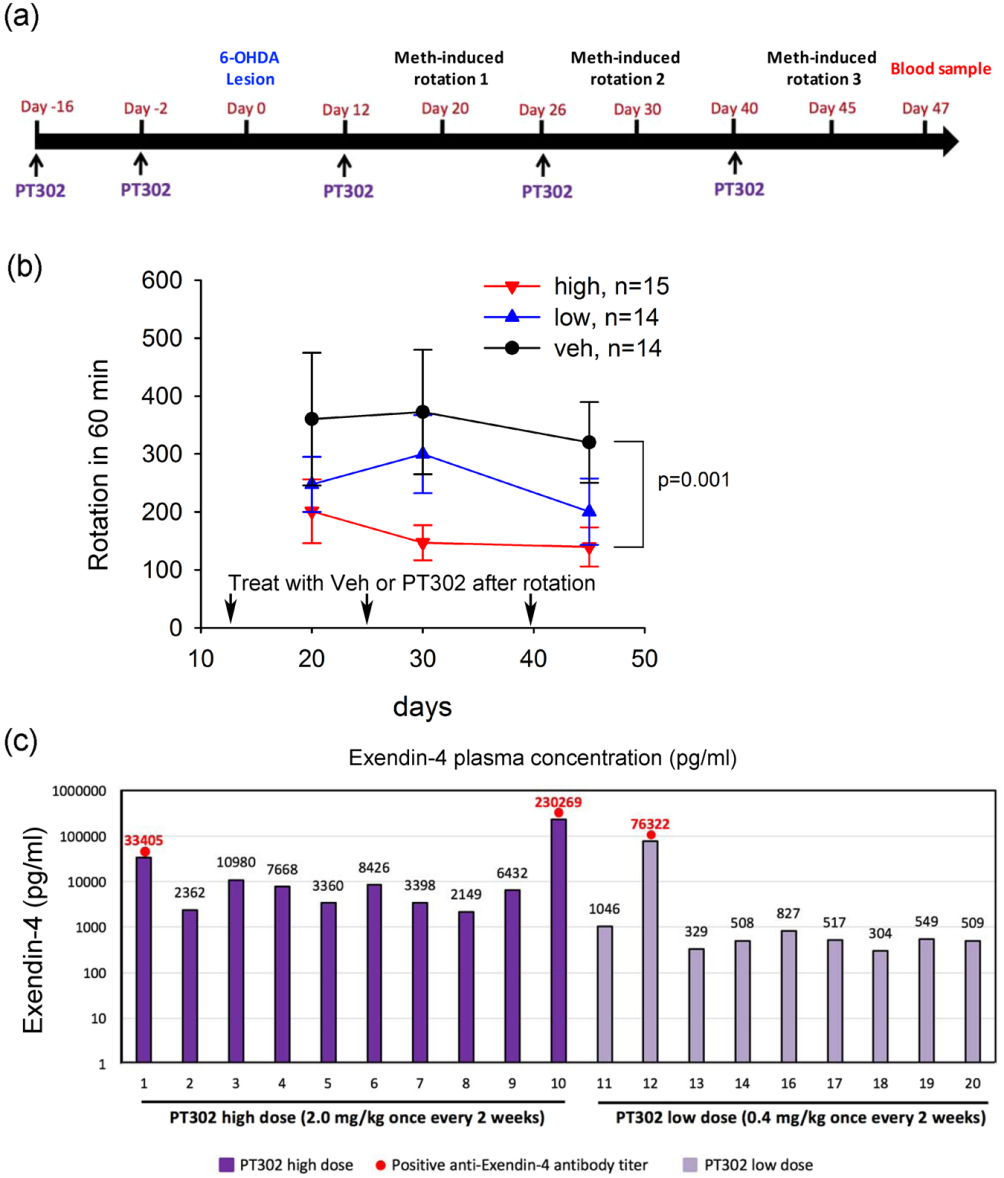
Pretreatment with PT302 reduces meth-mediated rotational behavior in hemiparkinsonian rats. Animals were treated with vehicle (n = 9), low dose PT302 (equivalent to Exendin-4 0.4 mg/kg every 2 weeks, n = 9) or high dose PT302 (equivalent to Exendin-4 2.0 mg/kg/ every 2 weeks, n = 10) starting 16 days prior to 6-OHDA lesioning. (**a)** Time line of study demonstrating PT302/vehicle dosing (day −16, −2, 12, 26 and 40 relative to day 0 when lesioning was performed), meth-mediated rotation was examined on days 20, 30 and 45 post-lesioning, a blood sample was taken on day 47 and animals were later euthanized. (**b**) Treatment with PT302 significantly reduced rotation (p = 0.018, F2, 87 = 4.309, [p < 0.001 in Fig. 2b] two way ANOVA). A post-hoc Newman-Keuls test indicated that the high dose of PT302 significantly attenuated meth-mediated rotation (p = 0.037). A non-significant decline was found between the vehicle and low dose PT302 groups (p = 0.156). (**c**) Exendin-4 plasma levels (pg/ml) were evaluated on day 47 for each animal, and were additionally evaluated for the presence of an anti-Exendin-4 antibody titer. Animals No. 1, 10 and 12 demonstrated a positive anti-Exendin-4 antibody titer (red circle).

As an initial evaluation of efficacy under idealized conditions, whereby dopaminergic cells within the brain would be challenged with a 6-OHDA insult in the presence of a potentially neuroprotective therapy, animals were pretreated with vehicle (9 rats), 0.4 mg/kg PT302 (low dose; 9 rats), or 2 mg/kg PT302 (high dose; 10 rats) 16 and 2 days prior to a 6-OHDA unilateral lesion of the medial forebrain bundle (Fig. 2a). Following the lesion, animals were again treated on days 12, 26 and 40, and subjected to meth-mediated rotation on days 20, 30 and 45 post-lesioning. A blood sample was collected for analysis of Exendin-4 levels on day 47 and animals were thereafter euthanized. Treatment with PT302 significantly reduced rotation (Fig. 2b) (p = 0.018, F_2,87_ = 4.309, two way ANOVA) relative to vehicle. A post-hoc Newman-Keuls test indicated that the high dose of PT302 significantly attenuated meth-mediated rotation (p = 0.037). A non-significant decline was noted in the low dose group (p = 0.156). Plasma levels of Exendin-4 were quantified from samples obtained on day 47 (Fig. 2c). Mean levels of Exendin-4 were 30.8 ng/ml (n = 10) and 8.99 ng/ml (n = 9) in the high and low dose groups, respectively. On removing values in which anti-Exendin-4 antibodies were found present [rats 1, 10 and 12 (Fig. 2c red circle values)], mean plasma Exendin-4 levels were 5.6 ng/ml (n = 8) and 574 pg/ml (n = 8) in the high and low dose groups, respectively.

In summary, biweekly administration of sustained release Exendin-4 in the form of PT302 significantly mitigated behavioral impairments induced by a unilateral 6-OHDA lesion of the medial forebrain bundle in rats, suggestive of neuroprotective activity.

### Post-treatment with PT302 reduces meth-induced rotational behavior in 6-OHDA lesion induced hemiparkinsonian rats

**Figure 3:**
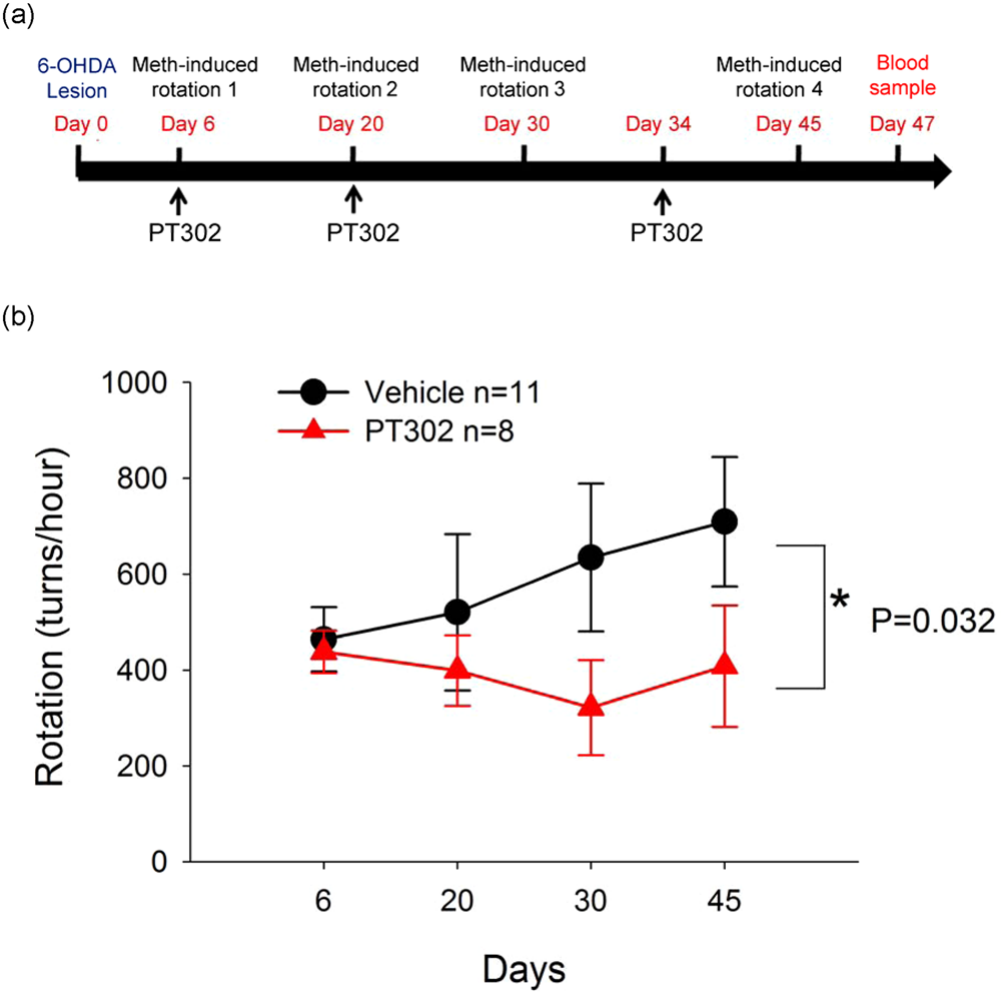
Post-treatment with PT302 reduces meth-mediated rotational behavior in hemiparkinsonian rats. Animals were treated with vehicle (n = 11) or high dose PT302 (equivalent to Exendin-4 2.0 mg/kg/ every 2 weeks, n = 8). (**a)** Time line of study demonstrating PT302/vehicle dosing (days 6, 20 and 34 relative to day 0 when lesioning was performed), meth-mediated rotation was examined on days 6, 20, 34 and 45 post-lesioning, a blood sample was taken on day 47 and animals were later euthanized. (**b**) Treatment with PT302 significantly reduced meth-induced rotation (p = 0.032, two way ANOVA).

Following positive behavioral indications of PT302-mediated neuroprotection as a pretreatment to a unilateral 6-OHDA lesion (Fig. 2b), the high PT302 dose (2 mg, s.c., biweekly) was evaluated as a post-treatment, as shown in Fig. 3a. In this scenario, rodents were challenged with a unilateral 6-OHDA lesion (day 0), and treatment was initiated 6 days thereafter in this more difficult to treat rodent model of PD, in which dopaminergic cell dysfunction and phenotype loss has already been initiated and is ongoing prior to treatment. Meth-induced rotation was also examined 6 days following the unilateral lesion. Before the initiation of PT302 treatment, animals that rotated in excess of 300 turns/hour were randomly separated into two groups to equalize group rotational behavior for vehicle (11 rats) or PT302 groups (8 rats). There was no difference between these groups: (p = 0.528, t-test). Meth-induced rotation was re-examined 20, 30 and 45 days after lesioning and was significantly reduced in the PT302 treatment group (F_1,67_ = 4.781, p = 0.032, two way ANOVA) compared to the vehicle controls (Fig. 3b). Notably, PT302 did not significantly alter body weight, as evaluated on day 45 (veh: 345 ± 6.5 g vs. PT302: 334 ± 9.8 g, p = 0.336, t-test).

### Post-treatment with PT302 protects against 6-OHDA–induced dopaminergic neurodegeneration in striatum

**Figure 4:**
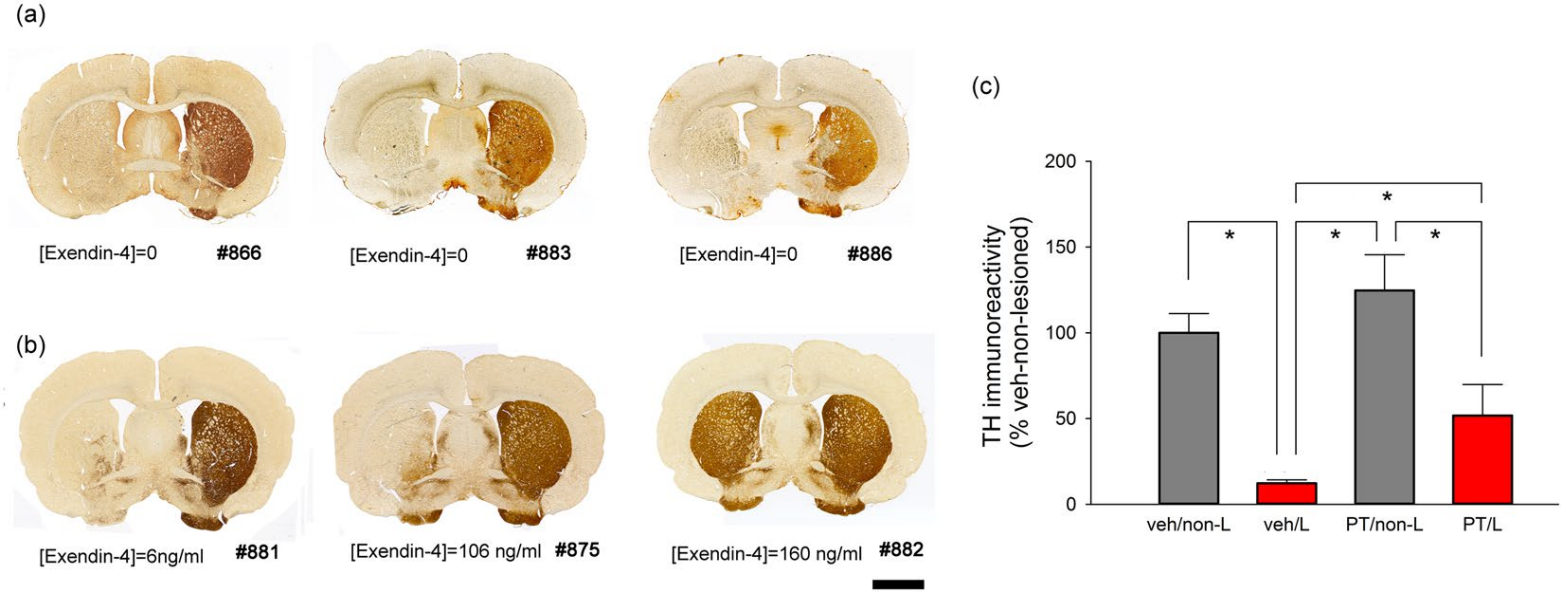
PT302 reduces 6-OHDA–mediated dopaminergic neurodegeneration in striatum. Typical striatal TH immunostaining and plasma Exendin-4 levels from (**a**) 3 rats (#866, 883, 886) receiving vehicle and (**b**) 3 rats (#881, 875, 882) receiving PT302. (**c**) Unilateral Injection of 6-OHDA significantly reduced striatal TH-IR in animals receiving vehicle (see left side). PT302 significantly mitigated TH-IR loss within the lesioned striatum (*p < 0.001, 2-Way ANOVA). L = lesioned side; non-L = non-lesioned side; veh = animals receiving vehicle; PT = PT302. Calibration = 250  μm.

Striatal tyrosine hydroxylase (TH) immunostaining from 3 representative rats receiving PT302 and vehicle is shown in Fig. 4a,b. Striatal TH-IR in brain sections with visible anterior commissure from 3 sections from each animal was averaged. 6-OHDA unilateral lesioning significantly reduced striatal TH-IR (p < 0.001, F_1,34_ = 36.784, 2-Way ANOVA) in animals receiving vehicle (p < 0.001, posthoc Newman-Keuls test) or PT302 (p = 0.001), comparing the lesion to the non-lesioned side (Fig. 4a,b). Notably, PT302, significantly mitigated the loss of TH-IR in the lesioned striatum (p = 0.001, posthoc Newman-Keuls test, Fig. 4c), as compared to vehicle. TH-IR within the non-lesioned side (contralateral) striatum was not affected by PT302 (p = 0.202, PT302 vs. vehicle, Fig. 4b,c). These data suggest that TH terminals within striatum were partially protected by PT302. Interestingly, there was a trend for greater preservation of TH fibers in animals with higher plasma Exendin-4 levels, as evident in Fig. 4b (rat #881, 6 ng/ml; E, rat #875, 107 ng/ml; F, rat # 882, 160 ng/ml).

### PT302 post-treatment protects against 6-OHDA–mediated dopaminergic neurodegeneration in substantia nigra

**Figure 5:**
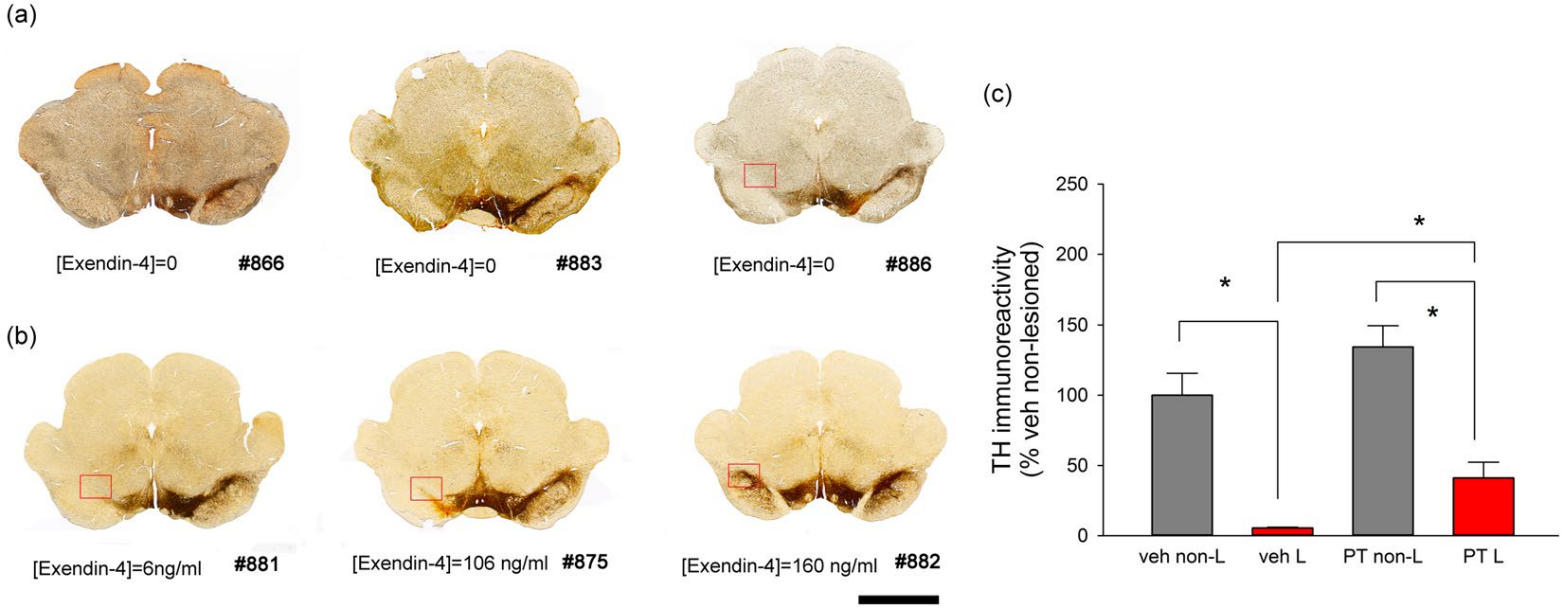
Post-treatment with PT302 protects against 6-OHDA–mediated dopaminergic neurodegeneration in the substantia nigra. Typical TH immunostaining from animals receiving vehicle (**a**), 3 rats (#866, #875, #886) or PT302 (**b**), 3 rats (#881, #875, #882). 6-OHDA lesioning caused a loss of TH-IR on the lesioned side in animals receiving vehicle (see left side). Treatment with PT302 ameliorated this TH-IR loss on the lesioned side. (**c**) TH-IR in substantia nigra was quantified every 360 um from bregma −4.2 mm to −6 mm for each animal. Post-treatment with PT302 significantly attenuated the loss of TH activity within the lesioned substantia nigra (*p < 0.001, 2-Way ANOVA. (L veh vs L PT302: *p < 0.001). Calibration = 250 μm.

TH-IR within midbrain brain sections was examined across all animals. Representative TH immunostaining from animals receiving vehicle (Rat #866, #875, #886) or PT302 (Rat #881, #875, #882) is shown in Fig. 5a,b. Unilateral 6-OHDA lesioning attenuated TH-IR within the substantia nigra in animals treated with either vehicle or PT302 (Fig. 5a,b), and treatment with PT302 mitigated this TH-IR loss. In those receiving vehicle, TH-IR in substantia nigra on the lesioned side was reduced to <10% of levels on the non-lesioned side. Post-treatment with PT302 significantly abated this decline in TH activity on the lesioned side (Fig. 5c, p < 0.001, 2-Way ANOVA), without affecting the contralateral (non-lesioned) side (p = 0.432).

**Figure 6:**
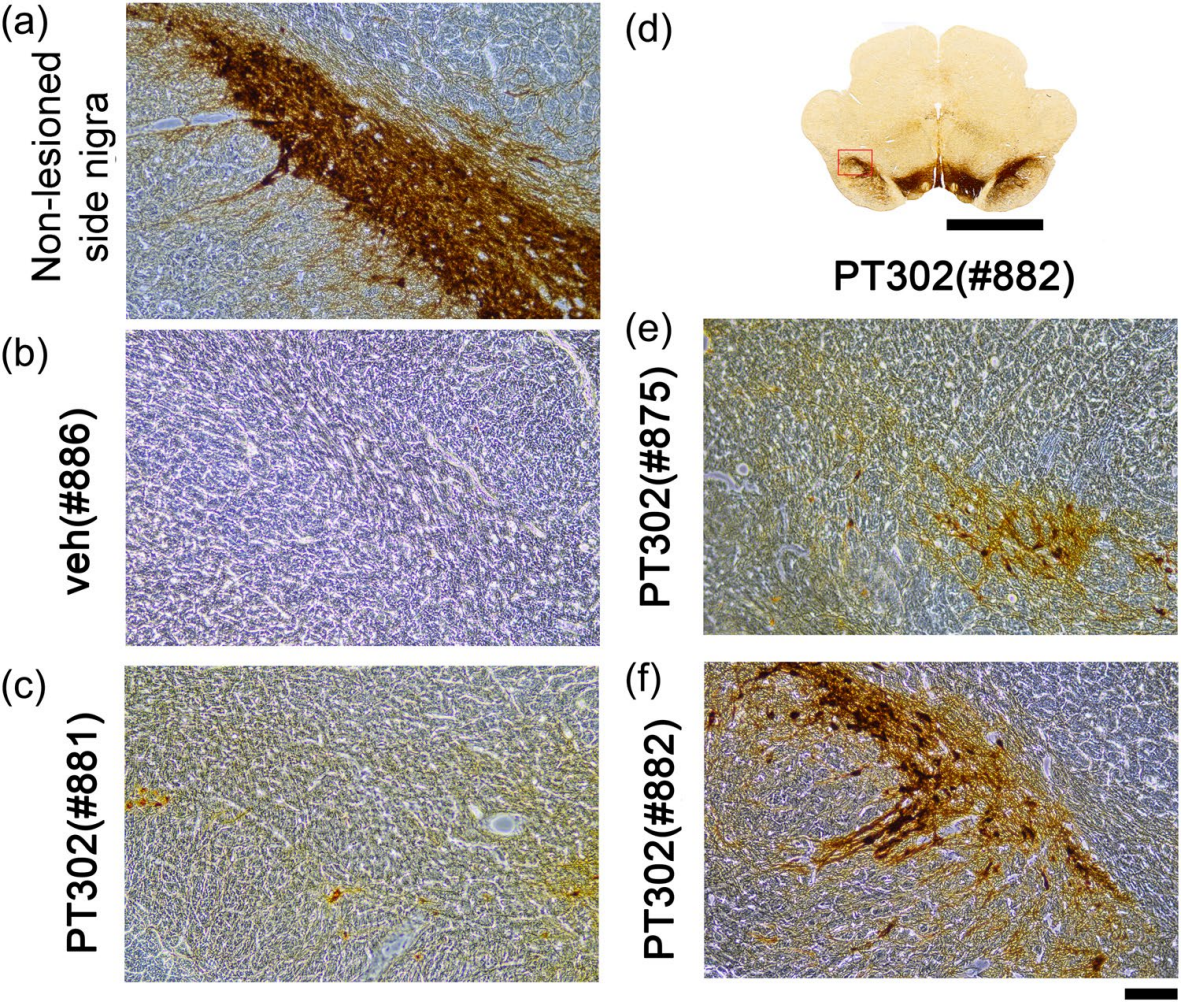
PT302 post-treatment protection of TH+ neurons within the 6-OHDA lesioned substantia nigra at higher magnification. (**a**) TH+ neurons were found on the non-lesioned side of substantia nigra across all animals. (**b**) Whereas almost no TH+ neurons or fibers were evident within the substantia nigra on the 6-OHDA lesioned side of vehicle-treated animals (typified by rat #886), post-treatment with PT302 partially protected TH+ neurons (**c**,**e**,**f** from rats #881, #8875 and #882, respectively). Calibration: (**d**): 250 μm; **a**,**b**,**c**,**e**,**f**: 100 μm.

As evident under higher magnification, almost no TH (+) neurons were found on the lesioned side in animals receiving vehicle (Fig. 6b, rat #886). In those administered PT302 (Fig. 6c: #881; E: #863; F: #882), TH (+) cells were partially protected. Similar to the results from the striatum (Fig. 4a,b), a greater preservation of TH-IR was evident in animals with high plasma Exendin-4 levels (Fig. 6c,e,f).

### Plasma Exendin-4 levels positively correlate with TH immunoreactivity

**Figure 7:**
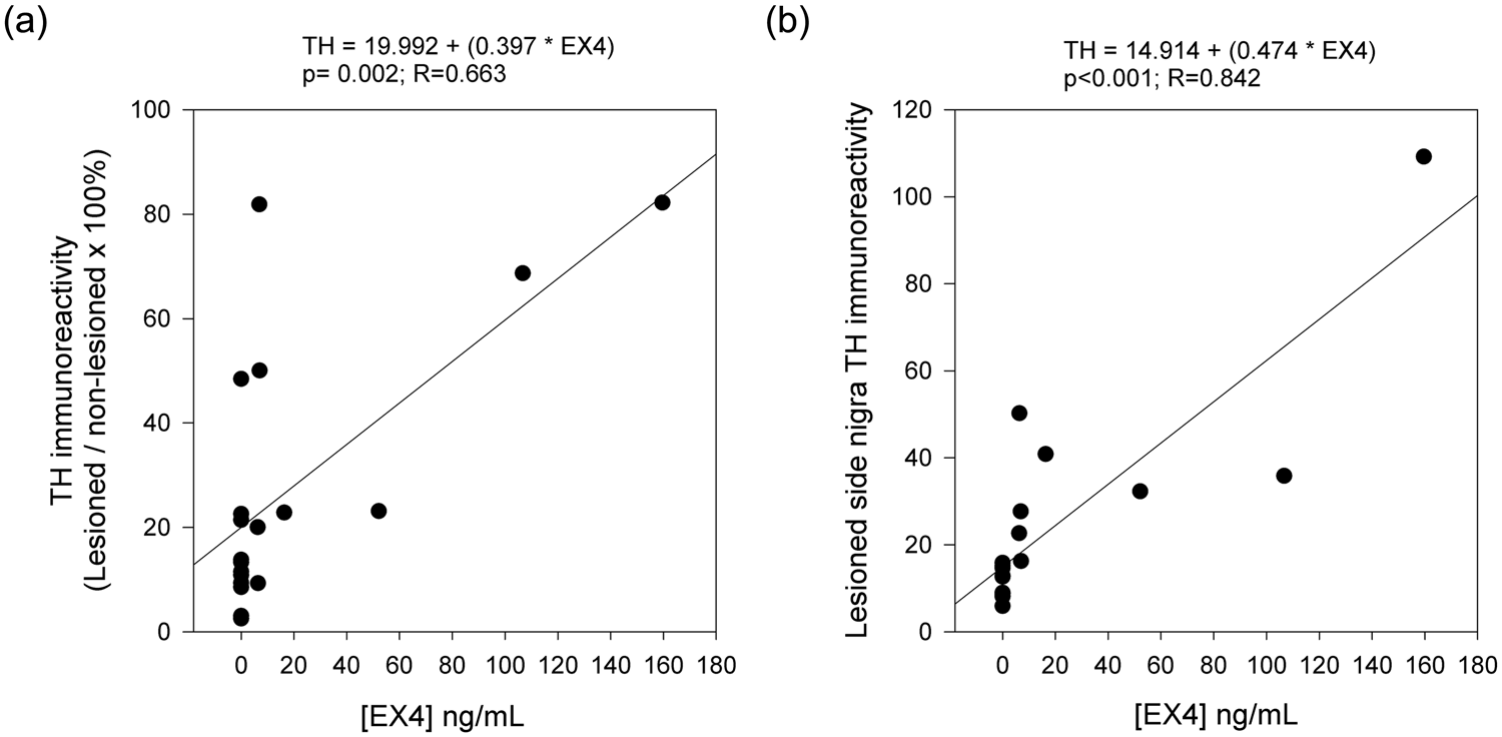
Exendin-4 mediated protection in TH neurons is associated with the plasma Exendin-4 level. (**a**) A significant positive correlation was found between normalized striatal TH-IR (i.e., lesioned/non-lesioned side) and plasma Exendin-4 levels (p = 0.002, R = 0.663). (**b**) Plasma Exendin-4 levels were significantly correlated with TH-IR in the substantia nigra on the lesioned side (p < 0.001, R = 0.842).

In light of the observed trend for greater preservation of TH fibers within the striatum, or neurons within the substantia nigra with higher plasma Exendin-4 levels (Figs 4b and 5b), we next examined the correlation between TH-IR and plasma Exendin-4 levels. TH-IR on the lesioned side of striatum was normalized to that on the non-lesioned side for each animal (i.e., lesioned ÷ non-lesioned value × 100%). A significant positive correlation was found between normalized striatal as well as substantia nigra TH-IRs and plasma Exendin 4 levels (striatum: Fig. 7a, p = 0.002, R = 0.663; substantia nigra: Fig. 7b, p < 0.001, R = 0.842). These data suggest that protective effects of PT302 for TH+ neurons are related to the plasma Exendin 4 levels achieved by PT302 treatment.

### Sustained release Exendin-4 enters the central nervous system

To evaluate the uptake of Exendin-4 into brain, particularly following its sustained release in the form of PT302, Exendin-4 levels were quantified in plasma and CSF samples 14 days following s.c. administration of a single dose of 0.46 mg and 2.0 mg. As shown in Table 1, the plasma/CSF ratio of Exendin-4 proved to be dose independent and was 1% of concomitant plasma levels. The s.c. administration of direct release Exendin-4 at doses that approached the plasma levels achieved by the sustained release PT302 formulation did not achieve quantifiable levels in CSF, and can hence be considered to be in the order of 6.9 pg/ml or less (the lower limit of detection of the ELISA).

**Table 1:** Plasma and Cerebral Spinal Fluid Levels in rats treated with Exendin-4 in the form of s.c. PT302 and immediate release Exendin-4

Formulation	Dose	Plasma (pg/ml)	CSF (pg/ml)	CSF/Plasma ratio
PT302(low)	0.46 mg/kg/14days	1853.3	18.3	0.01
PT302(high)	2.0 mg/kg/14days	2316.8	30.0	0.011
Exendin-4 low	2.3 µg/kg/day (1.15 BID)	93.5	Under LLOQ	N/A
Exendin-4 Medium	4.6 µg/kg/day (2.3 BID)	576.9	Under LLOQ	N/A
Exendin-4 High	10 µg/kg/day (5 BID)	5819.3	Under LLOQ	N/A

LLOQ: lower limit of quantification (6.9 pg/ml).

PT302 was administered as a one-time s.c. dose, and plasma and CSF were sampled exactly 14 days later.

Immediate release Exendin-4 was administered s.c. twice daily × 14 days, and plasma and CSF were sampled 90 min following the final dosing.

## Discussion

There is currently increasing interest in the value of GLP-1 receptor agonists as a neuroprotective treatment strategy for neurodegenerative disorders, and particularly for PD in the light of recent clinical trials with the T2DM approved drug, Exendin-4^35^, as well as ongoing and planned clinical safety/efficacy studies with liraglutide (NCT02953665) and lixisenatide. In a recently completed Exendin-4 randomized, double-blind, placebo-controlled trial, moderate stage PD patients administered once-weekly s.c. 2 mg Exenatide (*Bydureon*) over a 48 week duration, demonstrated a statistically significant reduction in the severity of their motor symptoms, as evaluated by the Movement Disorders Society Unified PD Rating Scale (MDS–UPDRS) motor subscale (part 3), which was the primary outcome measure of the study. PD subjects in the Exendin-4 group had an adjusted advantage of −3.5 points (with a lower score indicating less-severe symptoms) versus those in the control group (*P* = 0.0318) when their symptoms were investigated in the defined ‘Off medication’ state at 12 weeks following cessation of the Exendin-4 treatment. These encouraging results are suggestive of potential disease modifying, rather than symptomatic, effects and were achieved with a median CSF concentration of 11.7 pg/ml Exendin-4^35^. Whereas prior preclinical studies indicate that Exendin-4 can cross the blood–brain barrier and provide neuroprotective and regenerative effects across multiple animal models of PD^21,37^, no evidence is available from preclinical literature to show that doses similar to those used in patients provide meaningful effects on dopaminergic symptoms^38^. In the current study, we demonstrate that systemic administration of PT302, given once every two weeks by the s.c. route (as in humans), provides sustained plasma Exendin-4 levels that can be maintained for multiple weeks by repeated administration in the classical 6-OHDA unilateral medial forebrain bundle lesion model of PD. Post-treatment with PT302 reduced rotational behavior in hemiparkinsonian rats, and partially preserved dopaminergic terminals within the lesioned striatum and dopaminergic neurons in the lesioned substantia nigra. This was achieved at an Exendin-4 CSF concentration (18.4 to 30 pg/ml) within the range of that reported in recent clinical trials in PD patients^35^. Our data hence indicate that PT302 has neuroprotective actions for nigrostriatal dopaminergic neurons in the 6-OHDA rat model of PD, and provides an efficient method to support sustained plasma levels of Exendin-4 by once every two-week s.c. administration for future human studies.

Exendin-4, in the form of *Byetta* (immediate release, BID s.c.) and *Bydureon* (once weekly, biodegradable microspheres), is a licensed and efficacious drug for the long-term management of T2DM^39^. This GLP-1R agonist enhances glucose control by stimulating insulin release from pancreatic β-cells and inhibiting glucagon release. Dysfunctional insulin signaling consequent to the gradual occurrence of insulin resistance potentially triggers several common pathological processes that underpin both T2DM and neurodegenerative disorders, including PD^10,11,13,21^. Insulin regulates numerous biological processes within the brain, including energy homeostasis and neuronal survival, and facilitates its actions via insulin-receptor substrate-1 (IRS-1) that provide a gateway for the insulin signal to activate select downstream intracellular pathways, particularly the PI3K-Akt (phosphoinositide 3-kinase - serine/threonine kinase Akt (Protein kinase B) pathway) and the MAPK/ERK pathways (mitogen-activated protein kinases/extracellular signal-regulated kinases pathways)^21^. The former pathway provides access to the key downstream substrates, mTOR (mechanistic target of rapamycin), GSK3β (glycogen synthase kinase 3β) and FOX01 (forkhead box protein 01), which collectively regulate neuronal survival/apoptosis cascades, neurite outgrowth, mitochondrial function, autophagy and neuroinflammation^40,41^. The MAPK/ERK pathway plays a key role in synaptic plasticity^42^. Neuronal GLP-1R activation results in an almost immediate increase in intracellular cAMP (cyclic adenosine monophosphate), the activation of PKA (protein kinase A) and PI3K, and their downstream pathways, particularly the MAPK/ERK and PI3K-Akt pathways^23,37,43^. Together, these pathways can enhance cell survival and promote neuroprotection via their intracellular actions by stimulating calcium channels, augmenting protein synthesis, cellular proliferation, and mitochondrial biogenesis as well as inhibiting apoptosis, inflammation and protein aggregation^21,23,35,37^.

The activity of PT302 in the classical 6-OHDA unilateral medial forebrain bundle lesion model of PD in our current study, whether administered prior to or 6 days following the lesion, is in accord with prior studies demonstrating Exendin-4 mitigates markers of dopaminergic cell loss and motor impairments across multiple animal PD models. A toxin widely used as a research tool for PD preclinical research is MPTP, which, on metabolism to 1-methyl-4-phenylpyridinium, is selectively transported into the dopaminergic neurons and induces cellular dysfunction and death by inhibiting mitochondrial complex I^44^. MPTP administration to rats or mice induces a substantial loss of dopaminergic neurons, accompanied by an inflammatory response. GLP-1R agonists can protect against MPTP toxicity, with Exendin-4 fully reversing MPTP-induced losses in TH-IR, declines in dopamine and metabolite (3,4-dihydroxyphenylacetic acid and homovanillic acid) concentrations, motor impairments^26^ and neuroinflammation^45^. The neurotoxin 6-OHDA likewise induces dopaminergic cell dysfunction and apoptosis and similarly, GLP-1 and Exendin-4 have been shown to dose-dependently protect both immortal human SH-SY5Y neuronal cells that possess dopaminergic features, as well as rat primary ventral mesencephalic neuronal cultures that are rich in dopaminergic neurons, from 6-OHDA-induced cell death^26,43^. In rodents challenged with a 6-OHDA lesion, treatment with Exendin-4 mitigated declines in levels of TH-positive and vesicular monoamine transporter 2 (VMAT2)-positive cells, disturbances in motor function and augmented neurogenesis^27,46^. Notably, whereas mitigation of 6-OHDA-mediated toxicity was cross-validated by the use of Exendin-4^47,48^ as well as by a dual incretin agonist^28^, in other studies not all incretin mimetics have demonstrated activity in preclinical toxin-based PD models. Specifically, liraglutide failed to demonstrate efficacy in a nigral 6-OHDA partial or full lesion rat model^49^ and once daily Exendin-4 administration in an MPTP model failed to mitigate losses of TH-IR in substantia nigra or striatum, or motor impairments at the dose selected (10 nmol/kg once daily i.p.)^50^, albeit the GLP-1R agonists liraglutide (25 nmol/kg) and lixisenatide (10 nmol/kg) did demonstrate neuroprotection. In these studies, neither plasma nor CNS drug concentrations were measured and thus it is difficult to assess whether or not they achieved, or were maintained in, the therapeutic range. To our knowledge, there are currently no literature reports of GLP-1R agonists in α-synuclein rodent models of PD; however, the promising activity of Exendin-4 (administered by s.c. pump to maintain long-term steady-state levels) in a murine model of multiple system atrophy that involves the development of an α-synuclein load in the striatum^51^, suggests that the evaluation of long-term administration of Exendin-4 in PD α-synuclein models may represent a promising avenue. The question of providing histochemical correlates for functional changes in animal models of PD is important. However, both clinical and preclinical literature suggest a significant temporal disconnect between drug-induced functional changes in behavior and dopaminergic phenotype, the focus of this paper, and correlative changes in nigral dopaminergic neuron numbers. Of great significance is the study by Leenders *et al*.^52^ in late PD using PET that, at a stage where post-mortem examinations show an “extreme” depletion of DA, almost half the nigrostriatal dopamine cell numbers and striatal projections are still intact. Thus clinically there is a significant time lag between loss of dopamine together with PD symptoms compared to loss of dopaminergic cells.

There is also abundant literature in preclinical studies showing similar findings. Studies from the Zigmond lab^53^ and Stott and Barker^54^ indicate that the loss of dopaminergic phenotype occurs earlier than dopaminergic cell and fiber loss in 6-OHDA medial forebrain bundle -lesioned animals, the model used here. Moreover, work from Prezborski’s lab (summarized in Jackson-Lewis *et al*.^55^), using a genetic model, showed a similar loss of dopamine-mediated behavior despite only minimal changes in dopaminergic neurons. Given the considerations noted above, and a series of important studies from Burke’s lab^56^, we carried out correlative studies on behavior and TH nigro-striatal positivity as these would be more strongly related in the 6-OHDA model than dopaminergic cell bodies.

Another important issue is which of the various animal models of PD would be most useful for a paper on therapeutic development. One should use a model with a robust phenotype and a clearly defined starting point of dopaminergic pathway degeneration. In addition, the model should be well established as a platform already successfully utilized for other PD therapeutic approaches. These considerations have led to the decision to use the unilateral 6OHDA model with injection into the medial forebrain bundle, which provides a robust phenotype paralleling stage-3 PD and with the added advantage of rotational screening with dopaminergic agonists. The excellent review by Jenner^57^ summarizes these considerations.

Studies indicate that incretin mimetics, similar to other drug classes, have concentration-dependent actions as well as different brain uptakes. Whereas GLP-1 and Exendin-4 have been demonstrated to enter the brain by simple diffusion^58,59^, few of the studies evaluating incretin mimetics in PD or other neurodegenerative preclinical models have evaluated pharmacokinetic measures in either plasma or brain. Our current study indicates that s.c. PT302 administration to rats provides sustained release of Exendin-4 into plasma, in line with PT302 in human studies^36^. In both species, Exendin-4 release from PT302 exhibited a biphasic pharmacokinetic profile, with an initial early plasma peak occurring within hours of administration, and sustained release ensuing within 7 days. A second higher plasma peak arises at approximately 14 days (Tmax), and quantifiable levels in plasma last in excess of 24 days. Higher doses maintained this profile and resulted in a linear rise in Cmax and AUC, also in accord with human PT302 use^36^. Notably, the initial regulated release of Exendin-4 into plasma achieved a concentration that was lower than the final Cmax. This has relevance to tolerability in humans, in which an initial sharp rise in Exendin-4 levels has been associated with nausea^60^. Exendin-4 levels in CSF were 1% of concomitant plasma levels (Table 1) when evaluated under steady-state conditions, achieved by PT302 administration. This compares favorably with our determined value of 2% in human PD patients administered Exendin-4 as *Bydureon*^35^. Interestingly, however, Exendin-4 levels were below the level of assay detection (6.9 pg/ml), when rats were given immediate release Exendin-4 BID for 14 consecutive days s.c. and evaluated at 90 min (the approximate Tmax of immediate release s.c. Exendin-4^61^). Such an administration protocol mimics the use of Exendin-4 in the form of *Byetta* in humans, whereby therapeutic concentrations are maintained for a limited number of hours each day; use of Exendin-4 in this form is hence considered a “short-acting” GLP-1R agonist^62^ consequent to its elimination half-life of 2.4 hr^61^. Although the initial clinical evaluation of Exendin-4 in PD was performed with *Byetta* in an open-label proof of concept clinical trial, and demonstrated efficacy^63^, and we and others have previously used immediate release Exendin-4 efficaciously in our preclinical studies^23,27,46^, our current study here suggests that the maintenance of sustained Exendin-4 levels in plasma from PT302 provides a driving force to achieve greater concentrations of peptide within the central nervous system (CSF Exendin-4: 18.3 to 30 pg/ml for PT302 vs. <6.9 pg/ml for immediate release Exendin-4 BID). Hence, sustained release formulations like PT302 are likely to be the preferable way of administering Exendin-4 for treatment of neurodegenerative disorders.

In the present study, successive dosing of PT302 resulted in a stable release of Exendin 4 over an extended duration (Supplemental Fig. 1). In studies involving 6-OHDA lesioning, blood samples were collected 47 days after the lesion, coinciding with 7 and 13 days after the final PT302 dose in pre- and post-lesion treatment studies, respectively. This allowed evaluation of how plasma Exendin-4 levels impacted treatment outcome measures within each animal. Significant positive correlations were evident between plasma Exendin-4 levels and TH-IR within the striatum and substantia nigra (Fig. 7a and b, respectively).

Administration of meth or other amphetamine analogs causes ipsilateral rotation in unilaterally 6-OHDA-lesioned rats due to a differential preservation of dopaminergic terminals on the intact side. The degree of ipsilateral rotation is related to the level of depletion of dopamine in the nigrostriatal pathway^64^. We also found a marginal correlation trend toward significance between rotational behavior on day 45 and normalized striatal TH-IR in animals receiving post Exendin −4 treatment (p = 0.067, R = 0.429, data not shown). TH terminals within the striatum and TH+ cell body phenotypes in the substantia nigra were both significantly preserved after PT302 treatment (Figs 4c and 5c), concordant with declines in Meth-induced rotation and rises in plasma Exendin-4 levels. Of note, the concentration range of plasma Exendin-4 evident after serial PT302 dosing on day 47 (Fig. 7) was far broader than that measured following an equivalent single dose (Fig. 1b), with animal #882 achieving an unusually high plasma Exendin-4 level of 160 ng/ml (Figs 4b and 5b). This animal demonstrated a positive anti-Exendin-4 antibody titer, as did samples associated with a high plasma Exendin-4 concentration shown in Fig. 2c (animal #1, 10 and 12). The presence of an anti-Exendin-4 antibody titer was, likewise, noted in those animals with an unusually high plasma level of Exendin-4 in the recent *Bydureon* PD human clinical trial^35^ but, similar to our current study, was not associated with a loss of Exendin-4 biological activity. Prior human studies have demonstrated that antibody development to s.c. administered therapeutic peptides, such as GLP-1R agonists and insulin, is not uncommon^65^. Low titers (≤125) occur in humans with T2DM given Exendin-4 formulated for BID as well as once weekly administration, 32% and 45% of patients, respectively, with 24 to 30 weeks of treatment, and do not appear to affect efficacy. Higher-titer antibodies (≥625), which were not found in our present study, were less frequent, 5% and 12% of patients, respectively, and may attenuate Exendin-4 efficacy^65^. Notably, such antibodies do not cross-react with human GLP-1 or glucagon, or impact the safety profile of Exendin-4 formulations^65^.

## Conclusion

PT302 s.c. administration provided sustained levels of Exendin-4 that can be maintained with biweekly serial dosing over an extended duration. The administration of Exendin-4 as a sustained release formulation results in greater brain penetration than does BID administration of immediate release Exendin-4 (CSF Exendin-4: 18.3 to 30 pg/ml for PT302 vs. <6.9 pg/ml for immediate release Exendin-4 BID). Clinically relevant PT302 doses, evaluated by Exendin-4 levels in plasma and CSF, reduced the severity of a 6-OHDA unilateral medial forebrain bundle lesion as assessed by meth-induced rotational behavior, loss of TH+ cells in the substantia nigra, and TH+ immunoreactivity in the striatum. This was seen whether PT302 was initially administered prior to or 6 days following the lesion. Hence PT302 has neuroprotective actions in a well-characterized toxin model of PD and may, therefore, be clinically useful for treating PD patients.

## Methods

### Animals

Adult male Sprague-Dawley rats (2 months old upon arrival) were used for this study. The use of animals was approved by the Animal Care and Use Committee of either the National Health Research Institute, Miaoli, Taiwan (NHRI-IACUC-102102-A), the Intramural Research Program, National Institute on Aging, Baltimore, MD, USA (protocol No. 331-TGB-2018) or Peptron Inc., Daejeon, Republic of Korea (AEC-20080430-0004). All animal experiments complied with the Animal Research: Reporting of *In Vivo* Experiments (ARRIVE) guidelines and were carried out in accordance with the National Institutes of Health guide for the care and use of Laboratory animals (NIH Publications No. 8023, revised 1978). Animals were single or double caged and provided with food and water ad libitum.

### Drugs and reagents

PT302 (Peptron Inc., Daejeon, Republic of Korea), a sustained release formulation of Exendin-4, contains a mixture of polymers (98%) and Exendin-4 (2%). For all studies, PT302 was freshly dissolved in diluent, maintained at 4 °C on wet ice, vortexed immediately prior to each administration and subcutaneously injected. The composition of the diluent used to prepare the PT302 suspension was 0.5% carboxymethylcellulose sodium, 5.0% D-mannitol and 0.1% Tween 80 (pH 6.66) in sterile, double distilled water. The dose of PT302 evaluated in pharmacokinetic studies involved the following Exendin-4 amounts: 2.0, 2.4, 4.8 and 9.6 mg/kg. PT302 was administered once every two weeks. The doses of PT302 selected for prior administration in the 6-OHDA unilateral lesion study contained 0.4 and 2 mg/kg Exendin-4 administered every two weeks, as illustrated in Fig. 2a, specifically on days 16 and 2 prior to the lesion and on days 12, 26 and 40 after the lesion. The PT302 dose selected for the post 6-OHDA unilateral lesion treatment study contained 2 mg/kg Exendin-4 similarly administered every two weeks, as shown in Fig. 3a (given on days 6, 20, and 34 after 6-OHDA lesioning). Vehicle control animals received the same volume of diluent (without Exendin-4, s.c.) on the same dosing schedule. In pharmacokinetic studies, time-dependent blood samples were obtained from rats from 0 hr up to 31 days (Fig. 1a, N = 6 per time point). In 6-OHDA lesion studies, a blood sample was obtained on day 47 after lesioning, and animals were later euthanized and the brains immediately frozen to −80°C. These blood and brain samples were stored (−80°C) for later plasma Exendin-4 and brain TH-IR measurement. Finally, in separate studies to evaluate the uptake of Exendin-4 into the central nervous system, unlesioned rats (N = 5/group) were given either (i) PT302 (s.c.) containing either 0.46 mg or 2.0 mg Exendin-4, or (ii) twice daily immediate release Exendin-4 (2.3, 4.6 and 10 ug/kg daily). Plasma and CSF (from cisterna magna) samples were obtained on day 14, which for immediate release Exendin-4 was at precisely 90 min following the final s.c. dose. These plasma and CSF pharmacokinetic studies were undertaken in unlesioned animals to ensure that Exendin-4 brain uptake was not potentially affected by a possible compromise in the blood-brain barrier at the 6-OHDA lesion site that, notably, is distant from the target dopaminergic areas (striatum and substantia nigra) evaluated in our study.

### Unilateral 6-OHDA lesioning

Rats were anesthetized with chloral hydrate (400 mg/kg, i.p.) and placed in a stereotaxic frame. 6-OHDA (2.76 µg/µl × 5 µl in 0.9% NaCl containing 0.2 mg/ml ascorbic acid) was unilaterally injected into the medial forebrain bundle (−4.4 mm AP, 1.2 mm ML relative to bregma and 8.4 mm below skull) over 4 min through a Hamilton microsyringe held by a stereotaxic arm. The microsyringe was lowered to the desired target locus within the brain using micromanipulators attached to the stereotaxic frame. The speed of injection (0.5 µl/min) was controlled by a syringe pump (Micro 4, WPI, Sarasota, FL). The needle was removed 5 min after the injection. A piece of bone wax was placed on the burr hole to prevent the leakage of fluid. The wound was sutured or clipped. Body temperature was monitored with a thermistor probe and maintained at 37 °C with a heating pad during anesthesia. After recovery from the anesthesia, body temperature was further maintained at 37 °C for 3 hr using a temperature controlled incubator. A total of 43 rats received unilateral 6-OHDA lesioning in the PT302 pre-treatment study, and 19 rats in the post-treatment study.

Our selection of chloral hydrate as an anesthetic agent for our studies was based the advantages of its (i) rapid onset of action, (ii) short duration of anesthesia, (iii) stable anesthetic plane, and (iv) maintenance of body temperature. Its disadvantages include an association with adynamic ileus (loss of GI motility with consequent fluid sequestration and constipation) in laboratory rodents. In the NIH Animal Program’s “Anesthesia Guidelines for Rodents”, chloral hydrate is specifically listed as an acceptable anesthetic for laboratory rodent surgery, provided that the concentration of drug is kept at 4% or lower, that the users provide an acceptable scientific justification for its use in preference to other rodent anesthetics, and that the animal(s) be kept under strict observation for any signs of adynamic ileus such as bloating or constipation.

### Meth-induced rotation

Rotational behavior^66^ was evaluated using an 8-channel rotometer system (RotoMax, AccuScan Instruments, Inc). Animals were challenged with meth (2.5 mg/kg) as previously described^67^ and illustrated in Figs 2a and 3a. In animals used to assess PT302 as a post-lesion treatment, Meth-induced rotation was evaluated on day 6, with those that rotated in excess of 300 turns/hour randomly separated into 2 groups to equalize group rotational behavior for vehicle or PT302 treatment. Meth-induced rotation was then re-examined on 20, 30 and 45 days after lesioning.

### Plasma and CSF levels of Exendin-4

Exendin-4 levels were quantified either by using the Peptron Exendin-4 EIA Kit (Peptron, Daejeon, South Korea) or the Exendin-4-Fluorescent Enzyme Immunoassay Kit (Phoenix Pharmaceuticals INC., Burlingame, CA). Each sample was evaluated in duplicate at a volume of 50 ul each, with a 1:10 dilution for plasma. Concentrations of Exendin-4 were thereafter determined from standard curves of newly prepared Exendin-4, following preliminary studies to ensure that all results fell within the linear range of the respective plasma and CSF standard curves. Notably, these Exendin-4 assays have no cross-reactivity with glucagon, oxyntomodulin, GLP-1 or GLP-2.

### Exendin-4 antibody measurement

The development of anti-Exendin-4 antibodies is not common in clinical studies, consequent to the low homology of Exendin-4 to native GLP-1. Since our studies used a slow-release form of Exendin-4 over an extended time, we evaluated plasma anti-Exendin-4 antibody levels using a homemade sandwich ELISA protocol. In brief, ELISA plates were coated with Exendin-4 at 4 °C overnight, and following blocking and washing steps, standards (mouse monoclonal anti-Exendin-4 antibody) and unknown samples were added to the plate and incubated at 37 °C for 1 h. After washing, biotinylated-Exendin-4 was added and followed by SA-HRP detection. The titers of the anti-Exendin-4 antibody within the samples were then estimated by serial dilution of the plasma (to a maximum dilution of 1:125).

### TH immunoreactivity

TH was examined by immunohistochemistry. Serial cryostat sections of the entire brain were cut at 25 μm thickness. One series from every sixth section was stained for TH. To control for variability, specimens from all experimental groups were included in each staining. Free-floating sections were rinsed in 0.1 M phosphate buffer (PB) and blocked/permeabilized with 4% bovine serum albumin (BSA) and 0.3% Triton x-100 in 0.1 M PB. Sections were then incubated in primary antibody (mouse monoclonal anti-TH diluted in 4% BSA and 0.3% Triton x-100 in 0.1 M PB, concentration 1:100; Chemicon, Temecula, CA) for 17–19 hours at 4 °C. Sections were then rinsed in 0.1 M PB and incubated in secondary antibodies for 1 hour, followed by incubation for 1 hour with avidin-biotin-horseradish peroxidase complex. Sections were mounted on slides, and coverslipped. Control sections were incubated without primary antibody and all observers were blinded as to treatment groups.

TH-IR in the striatum was measured by ImageJ software and was averaged from 3 brain sections with a visible anterior commissure. TH-IR in the substantia nigra was measured every 360 µm throughout the midbrain (from bregma – 4.2 mm to −6.0 mm). A total of 5 brain sections from each animal was used. The volume of the substantia nigra was analyzed using Cavalieri’s method.

### Statistical analysis

Values are expressed as means ± S.E.M. The Kolmogorov-Smirnov test was used to determine normality of distributions. Student’s t-test, Mann-Whitney tests, Fisher Exact test or 1- and 2-way ANOVAs were used for statistical analysis as indicated in results. ANOVA on ranks was used when the normality assumption was violated. Post-hoc Newman-Keuls test or Dunn’s test was used for all pairwise multiple comparisons. A statistically significant difference was defined as p < 0.05.

### Data Availability

The datasets generated during and/or analysed during the current study are available from the corresponding authors on reasonable request (contact: ywang@nhri.org.tw and/or greign@mail.nih.gov).

## Electronic supplementary material

Supplemental figure 1
