# Author Correction: Post-treatment with PT302, a long-acting Exendin-4 sustained release formulation, reduces dopaminergic neurodegeneration in a 6-Hydroxydopamine rat model of Parkinson’s disease

**DOI:** 10.1038/s41598-018-31455-w

**Published:** 2018-09-12

**Authors:** Shuchun Chen, Seong-Jin Yu, Yazhou Li, Daniela Lecca, Elliot Glotfelty, Hee Kyung Kim, Ho-Il Choi, Barry J. Hoffer, Nigel H. Greig, Dong Seok Kim, Yun Wang

**Affiliations:** 1 Center for Neuropsychiatric Research, National Health Research Institutes, Zhunan, Taiwan; 2 Graduate Institute of Applied Science and Engineering, Fu-Jen Catholic University, New Taipei, Taiwan; 3 Drug Design and Development Section, Translational Gerontology Branch, Intramural Research Program, National Institute on Aging, National Institutes of Health, Baltimore, MD USA; 4 Peptron Inc., Yuseong-gu, Daejeon, Republic of Korea; 5 Department of Neurosurgery, Case Western Reserve University School of Medicine, Cleveland, OH USA

Correction to: *Scientific Reports* 10.1038/s41598-018-28449-z, published online 16 July 2018

The original version of this Article contained a typographical error in the spelling of the author Dong Seok Kim, which was incorrectly given as Dong-Seok Kim. This has now been corrected in the PDF and HTML versions of the Article, and in the accompanying Supplementary Information file.

