# Supplementary material for: Post-treatment with PT302, a long-acting Exendin-4 sustained release formulation, reduces dopaminergic neurodegeneration in a 6-Hydroxydopamine rat model of Parkinson’s disease
Source: Sci Rep. 2018 Jul 16;8:10722. doi: 10.1038/s41598-018-28449-z (PMC6048117; doi:10.1038/s41598-018-28449-z)
Supplement: Supplementary file 1 — Supplemental figure 1 [file 41598_2018_28449_MOESM1_ESM.docx]

**Supplemental Title page**

**Manuscript title:** Post-treatment with PT302, a long-acting Exendin-4 sustained release formulation, reduces dopaminergic neurodegeneration in a 6-Hydroxydopamine rat model of Parkinson’s disease

Shuchun Chen ^1,2^, Seong-Jin Yu^1^, Yazhou Li^3^, Daniela Lecca^3^, Elliot Glotfelty^3^, Hee Kyung Kim^4^, Ho-Il Choi^4^, Barry J. Hoffer^5^, Nigel H. Greig^3^***^∆^**, Dong Seok Kim^3,4^**^∆^**, Yun Wang^1^***^∆^**

^1^ Center for Neuropsychiatric Research, National Health Research Institutes, Taiwan.

^2^ Graduate Institute of Applied Science and Engineering, Fu-Jen Catholic University, Taiwan

^3^ Drug Design and Development Section, Translational Gerontology Branch, Intramural Research Program, National Institute on Aging, National Institutes of Health, Baltimore, MD, USA.

^4^ Peptron Inc., Yuseong-gu, Daejeon, Republic of Korea.

^5^ Department of Neurosurgery, Case Western Reserve University School of Medicine, Cleveland, OH, USA.

**Supplemental Figure 1**

**Supplemental Figure 1**. Time-dependent Exendin-4 levels in plasma over a 10 week period in male Sprague-Dawley (9 week old) rats administered PT302 (equivalent to Exendin-4 2 mg/kg, s.c. QW (once weekly)) (n=6). Blood was collected prior to and at 1 and 3 hr after the initial PT302 dose, as well as on days 1, 3, 5, 7, 10, 14, 21, 28, 35, 42, 49, 56, 63, 70, 77, 84, and 91. Plasma levels of Exendin-4 were quantified as detailed in the Materials and Methods. The following parameters were determined: Cmax: 5.27ng/ml, Tmax: 51.60 days, and AUC of 236.42ng.d/mL.)
